# Anti-rituximab antibodies in patients with refractory autoimmune nodopathy with anti-neurofascin-155 antibody

**DOI:** 10.3389/fimmu.2023.1121705

**Published:** 2023-03-28

**Authors:** Yunfei Bai, Wei Li, Chuanzhu Yan, Ying Hou, Qinzhou Wang

**Affiliations:** ^1^ Department of Neurology, Qilu Hospital of Shandong University, Jinan, China; ^2^ Department of Central Laboratory and Mitochondrial Medicine Laboratory, Qilu Hospital (Qingdao), Cheeloo College of Medicine, Shandong University, Qingdao, China; ^3^ Brain Science Research Institute, Shandong University, Jinan, China

**Keywords:** anti-neurofascin 155 antibody, rituximab, anti-rituximab antibody, peripheral B cells, nodopathy

## Abstract

**Background:**

Recent studies have reported that similar to other IgG4 autoimmune diseases, such as muscle-specific kinase antibody-associated myasthenia gravis, most anti-neurofascin-155 (anti-NF155) nodopathies respond well to rituximab treatment, regardless of the dosage. However, there are still a few patients for which rituximab is ineffective for unknown reasons. Currently, there are no studies on the mechanism of ineffective treatment with rituximab.

**Methods:**

A 33-year-old Chinese man presenting with numbness, tremor, and muscle weakness for 4 years was recruited for this study. Anti-NF155 antibodies were identified by cell-based assay and confirmed by immunofluorescence assay on teased fibers. The anti-NF155 immunoglobulin (IgG) subclasses were also detected by immunofluorescence assay. Anti-rituximab antibodies (ARAs) were quantitatively analyzed using enzyme-linked immunosorbent assay (ELISA), and peripheral B cell counts were determined by flow cytometry.

**Results:**

The patient exhibited anti-NF155 IgG4-antibody positivity. After the first round of rituximab infusion, the patient showed stratified outcomes with improvements in numbness, muscle weakness and ambulation. However, after three rounds of rituximab infusion, the patient’s symptoms deteriorated, and the numbness, tremor and muscle weakness returned. No obvious improvement was found after plasma exchange and another round of rituximab treatment. 14 days after the last treatment with rituximab, ARAs were detected. And the titers gradually decreased on day 28 and 60 but remained higher than normal. Peripheral CD19^+^ B cell counts were less than 1% within the 2-month period following the final rituximab administration.

**Conclusions:**

In this study, ARAs presented in a patient with anti-NF155 nodopathy undergoing rituximab treatment and showed an unfavorable impact on rituximab efficacy. This is the first case to report the occurrence of ARAs in patients with anti-NF155 antibodies. We suggest that ARAs should be tested early during the initial intervention, especially in patients who respond poorly to rituximab treatment. In addition, we believe it is necessary to investigate the association between ARAs and B cell counts, their effect on clinical efficacy, and their potential adverse reactions in a larger cohort of patients with anti-NF155 nodopathy.

## 1 Introduction

Autoimmune nodopathies are characterized by antibody formation against nodal-paranodal cell-adhesion molecules such as neurofascin 155 (NF155), contactin 1 (CNTN1), contactin-associated protein 1 (Caspr1), and neurofascin 140/186 (NF140/186) (1). Unlike typical chronic inflammatory demyelinating polyneuropathy (CIDP), patients with these antibodies generally have specific clinical features such as tremor, sensory ataxia, and significantly elevated cerebrospinal fluid (CSF) protein. The nerve injuries occur due to the nodal-paranodal region’s dismantling. Segmental demyelination is absent, and the pathogenic mechanism is not inflammation-related (2). Therefore, autoimmune nodopathy is now classified as a separate entity rather than a subgroup of CIDP based on the latest criteria (1). Anti- NF155 nodopathy is regarded as a subgroup of autoimmune nodopathy. Since the most common isotype of anti-NF155 antibodies is immunoglobulin G4 (IgG4), anti-NF155-positive patients generally respond poorly to intravenous immunoglobulin (IVIg) therapy (3). Similar to other IgG4 autoimmune diseases, such as muscle-specific kinase antibody-associated myasthenia gravis, most anti-NF155 nodopathies respond well to rituximab treatment, regardless of the dosage (4–7). However, there are still a few patients for which rituximab is ineffective for unknown reasons (7). As rituximab is a human/mouse chimeric anti-CD20 monoclonal antibody with high immunogenicity, ARAs may be produced and lead to a decline of rituximab efficacy. As such, ARAs have been described in many autoimmune diseases such as CIDP, neuromyelitis optica spectrum disorder (NMOSD), systemic lupus erythematosus (SLE), and rheumatoid arthritis (RA) (8–12). ARAs might affect the pharmacodynamics of rituximab since ARA-positive patients often have a higher frequency of rituximab reinfusion, a higher rate of relapse, and a faster B cell reconstitution than ARA-negative patients (9, 13, 14). However, detailed information about ARAs in anti-NF155 nodopathy is scarce. In the present study, we report the presence of ARAs in a male patient with anti-NF155 antibodies who responded poorly to rituximab after five rounds of rituximab infusion and whose CD19^+^ B cell counts were below 1% within 2-months after the last rituximab treatment.

## 2 Materials and methods

### 2.1 Patient history and clinical data

A 33-year-old Chinese man presented to our hospital with a 4-year history of progressive numbness, tremor, and muscle weakness. From 29 years of age, the patient experienced numbness, unsteady gait, inability to squat, and tremor in both upper extremities. The patient received a diagnosis of CIDP by their primary healthcare provider in 2017 and was prescribed IVIg, corticosteroid, and azathioprine therapies; however, poor effect of these treatments was obtained on the patient’s symptoms, and the patient eventually loss of ambulation. In 2018, he received the first round of 200 mg IV rituximab and achieved marked clinical improvement 1 month later; he could walk and squat independently, and the numbness and tremor had improved. In 2019, the patient received a second round of 200mg rituximab administration to maintain the low B-cell counts. Three months later, he experienced a progressive deterioration in his clinical condition and received an increased dosage of 500mg rituximab treatment. Peripheral CD19^+^ B-cell counts were below 1% after these two rounds of rituximab treatment. However, the patient’s symptoms got deteriorated by mid-2020. He experienced unsteady gait, difficulty walking up and down steps, and a severe tremor in both hands. Intriguingly, peripheral CD19^+^ B cell counts had improved to 11.04%. A fourth round of 500 mg rituximab was administered, but the patient’s symptoms continued to worsen, and CD19^+^ B cell count went down to 2.58% 1-month after treatment. In late 2020, the patient was treated with 5 rounds of plasma exchange, but no clinical improvement was observed. In 2021, he was admitted to our hospital presenting with muscle weakness, numbness, and tremor. A fifth round of 500 mg rituximab was administered. However, there was still no improvement in clinical symptoms.

### 2.2 Physical examination

On admission to our hospital, neurological examination revealed no significant deficits in mental health or cranial nerves. The patient’s proximal upper and lower limbs and distal limbs were graded as 5 and 4, respectively. Deep-tendon reflexes were absent. Sensory examination revealed a glove and stocking hypoesthesia with impaired vibratory sensation in the finger and toe joints. Intentional tremor was observed. The Romberg and bilateral heel-knee-shin tests were both positive. The pyramidal and meningeal irritation signs were both negative. Hughes Disability Scale and modified Rankin scale (mRS) scores were 2 and 3, respectively.

### 2.3 Serum ARA ELISA

Samples of blood (~ 4 ml) were collected at admission and on days 1, 3, 7, 14, 28, and 60 after the final round of rituximab infusion in two anti-NF155-positive patients. Case 1 was the patient mentioned above in this study. Both patients received 500 mg rituximab. The other patient, defined as case 2 who does not receive rituximab administration before, got significant clinical improvement. A healthy individual was also recruited as a control. All samples were stored at -80°C prior to analysis. According to the manufacturer’s instructions, ARAs were measured using an enzyme-linked immunosorbent assay (ELISA) kit (Matriks Biotek, US).

### 2.4 Immunofluorescence assay on teased fibers

Teased fibers were dissected from the sciatic nerves of adult C57BL/6J mice, placed on adhesion microscope slides, and fixed in acetone at room temperature for 10 min. The slides were permeabilized with 1% Triton X-100 at 37°C for 30 min, blocked, and incubated with sera (diluted at 1:10 in PBS) and chicken anti-human/mouse/rat neurofascin antigen affinity-purified polyclonal antibodies (1:50; R&D Systems, Minneapolis, MN, USA) overnight at 4°C. They were then incubated with AffiniPure goat anti-human IgG Fcγ (1:200; Alexa Fluor 594; Jackson ImmunoResearch) together with AffiniPure goat anti-chicken IgY (IgG) (H+L) (1:200; Alexa Fluor 488; Jackson ImmunoResearch) at 37°C for 45 min. Images were acquired using a fluorescence microscope (Leica).

### 2.5 Antibody detection

In both the serum and the CSF, anti-NF155 autoantibody and its subclasses were detected using a cell-based assay (CBA) method. Specifically, the human NF155 coding sequence (NM_001160331) was subcloned into the pcDNA3.1 plasmid, and transfected into HEK293T cells. After 48 h, cells were fixed with cold acetone. The fixed cells were incubated first with patients’ serum (diluted with PBS) or CSF for 1 h and then incubated with the corresponding fluorescein isothiocyanate (FITC)-labeled secondary antibodies (FITC-goat anti-human IgG Fcγ. antibody (109-095-170, Jackson ImmunoResearch, PA, USA) for IgG detection; FITC-goat anti-human IgG1 (F0767, Sigma-Aldrich, MO, USA), IgG2 (F4516), IgG3 (F4641), and IgG4 (F9890) antibodies for IgG subclass detection) at 1:200 dilution for 30 min. Autoantibody reactivity was examined using a fluorescence microscope (Leica).

### 2.6 Clinical, neuroimaging, and electrophysiological data analyses

To evaluate the response to treatments, Hughes disability scale was used to assess the severity of disability in patients. We defined treatment responses in terms of △Hughes (the scale value after treatment minus the scale value before treatment) as follows: △Hughes < 0, effective; △Hughes = 0, with subjective or objective improvement, partially effective; and △Hughes>0, without any improvement, non-effective (15). Magnetic resonance imaging (MRI) of the brachial plexus and lumbosacral plexus were acquired using a 3.0 T MR scanner. The electrophysiological data characteristics involved motor and sensory conduction in the upper and lower extremities. Motor distal latency, motor conduction velocity, and amplitude were recorded bilaterally using standard protocols. We calculated the terminal latency index (TLI) following the method previously described by Katz et al. (16) as follows: TLI = distal distance/(proximal conduction velocity × distal latency).

## 3 Results

### 3.1 Antibody detection

**Figure 1:**
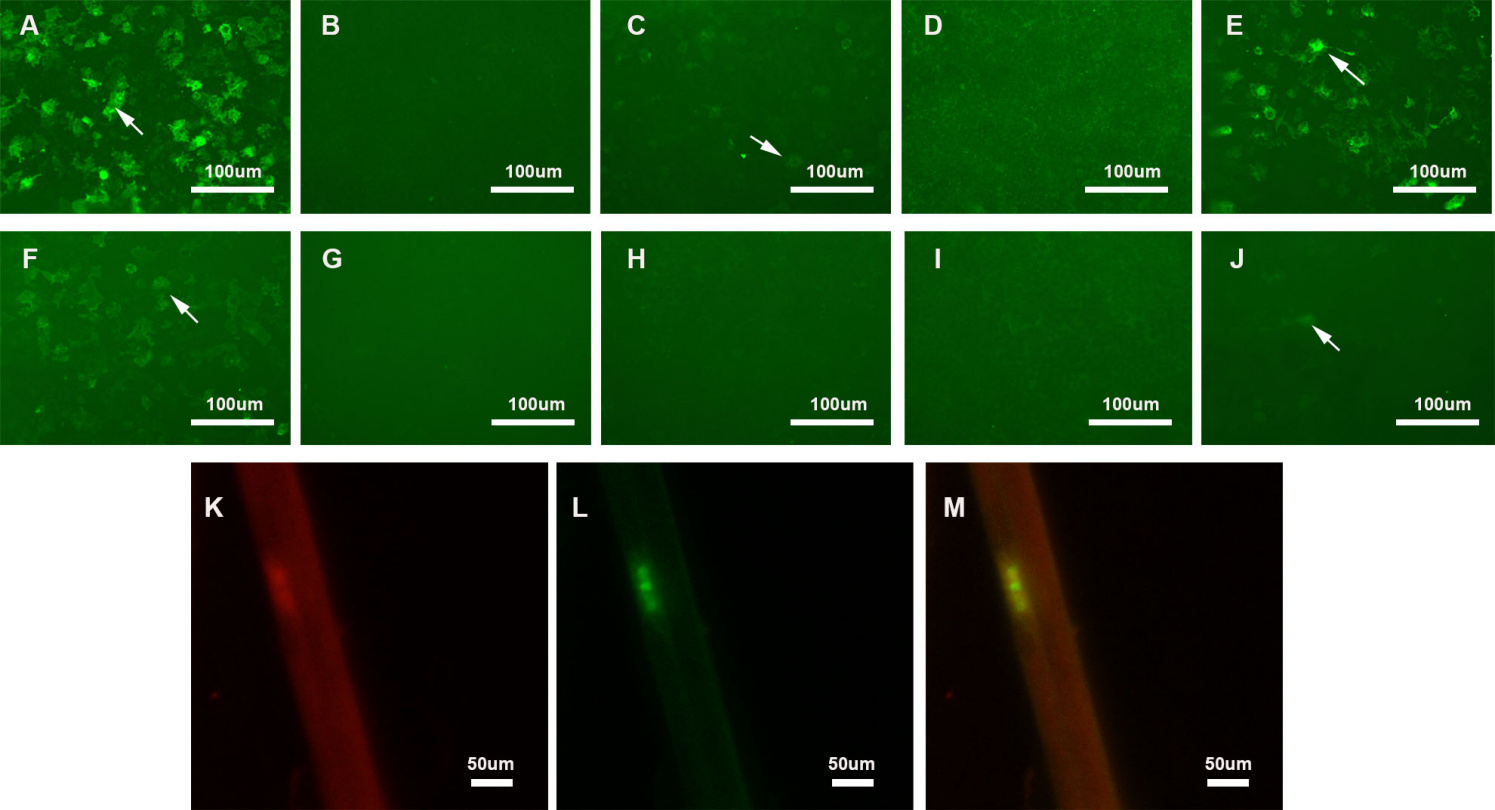
Detection of anti-NF155 antibodies. **(A–E)** Detection of anti-NF155 antibodies in serum. **(A)** 1:100 positive anti-NF155 antibodies (arrow); **(B)** Negative anti-NF155 IgG1 antibodies; **(C)** 1:10 positive anti-NF155 IgG2 antibodies (arrow); **(D)** Negative anti-NF155 IgG3 antibodies; **(E)** 1:32 positive anti-NF155 IgG4 antibodies (arrow); **(F–J)** Detection of anti-NF155 antibodies in CSF. **(F)** 1:3.2 positive anti-NF155 antibodies (arrow); **(G)** Negative anti-NF155 IgG1 antibodies; **(H)** Negative anti-NF155 IgG2 antibodies; **(I)** Negative anti-NF155 IgG3 antibodies; **(J)** 1:1 positive anti-NF155 IgG4 antibodies (arrow); **(K–M)** Double-immunofluorescence of murine teased fibers with serum sample of the patient. Seropositive of serum from Case 1 (Red). Optimal colocalization is noted in the paranodal region. (yellow). Scale bars = 100 μm **(A–H)**; Scale bars = 50 μm **(K–M)** NF155, neurofascin-155; CSF, cerebrospinal fluid; NF186, neurofascin-186.

Anti-NF155 IgG was 1:100 positive in serum and 1:3.2 positive in CSF using the CBA method. We confirmed that the serum showed reactivity against the paranodal region using an immunofluorescence assay on teased fibers of murine sciatic nerves. In addition, analysis of the IgG subclasses demonstrated that our patient had 1:32 and 1:1 positive anti-NF155 IgG4 in the serum and CSF, respectively. The patient also had 1:10 positive anti-NF155 IgG2 in the serum (**Figure 1**). Other nodal, paranodal, and juxtaparanodal antibodies, including anti-NF186 antibody, anti-Caspr1 antibody, and anti-CNTN1 antibody, were negative in the serum and CSF.

### 3.2 Laboratory findings and cerebrospinal fluid test

Laboratory tests revealed an erythrocyte sedimentation rate of 27 mm/h. Other blood analyses for testing liver and kidney function, C-reactive protein, anti-nuclear antibodies, thyroid function, syphilis serology, and HIV were all within the normal ranges. Lumbar puncture was performed with a significantly elevated protein level of 1.45 g/L and a leukocyte count of approximately 2/mm^3^. Common encephalitis-causing pathogens were not detected.

### 3.3 Electromyography and MRI findings

**Figure 2:**
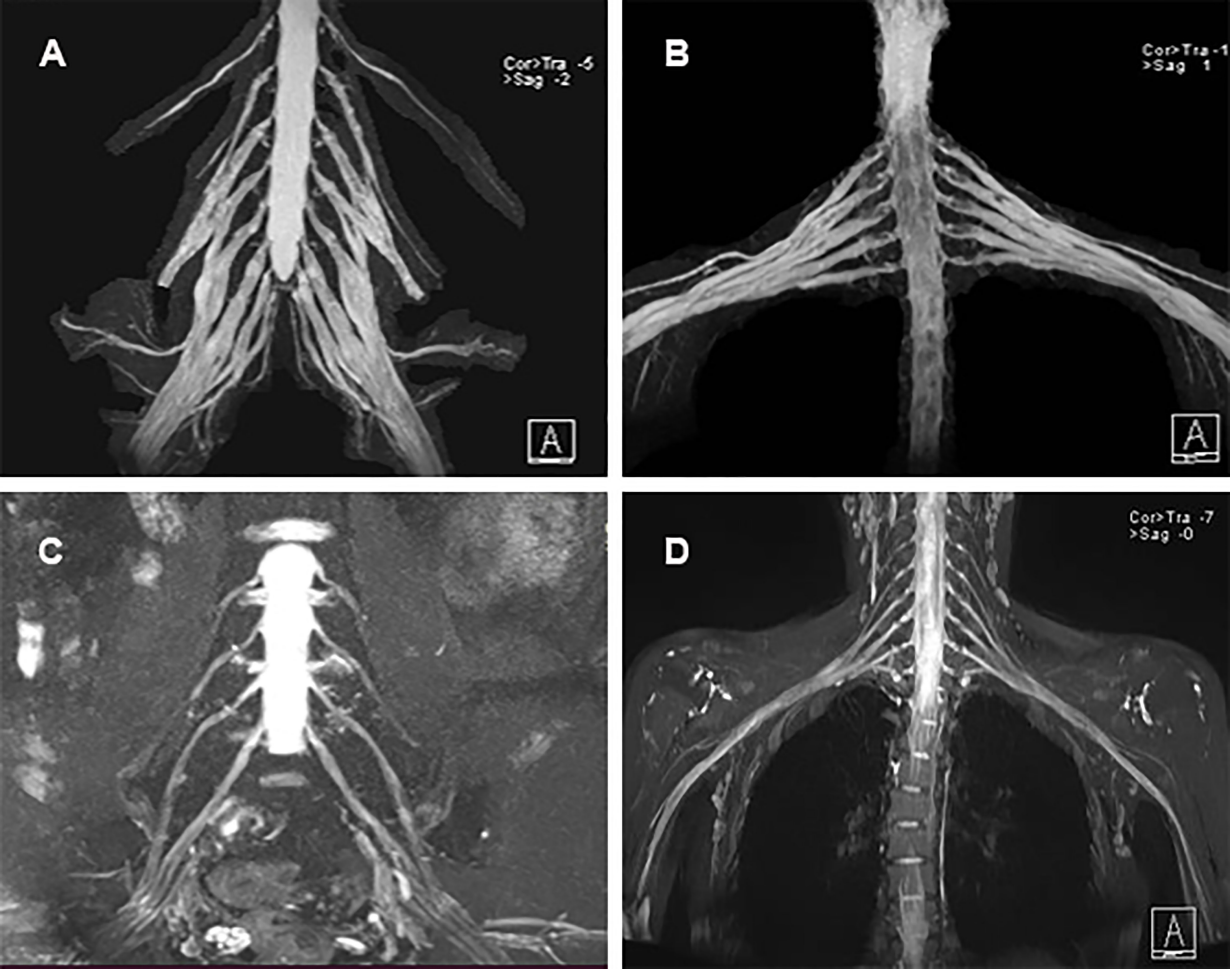
MRI neurography findings. **(A)** Diffuse thickening of the cervical roots; **(B)** Diffuse thickening of the lumbosacral nerve roots; **(C, D)** Normal cervical roots and lumbosacral nerve roots of healthy control.

Electrodiagnostic studies showed reductions in motor conduction velocity and motor nerve amplitude. Sensory nerve action potentials were absent in both the upper and lower extremities, and distal motor latency was prolonged (**Table 1**). The sympathetic skin response (SSR) also showed abnormalities. MRI revealed marked symmetric hypertrophy of cervical and lumbosacral roots. Diffuse thickening of the bilateral intercostal nerves were also observed (**Figure 2**). The brachial plexus and lumbosacral plexus did not show contrast enhancement.

**Table 1:** Detailed electrophysiologic data of our patient with ARAs.

	Left	Right
MOTOR		
Ulnar nerve		
DML, ms	5.83	6.71
CV, m/s	16.0	17.1
CMAP amplitude	4.7	3.5
TLI	0.536	0.436
Median nerve		
DML, ms	7.71	8.17
CV, m/s	16.0	20.1
CMAP amplitude	3.7	3.4
TLI	0.486	0.365
Tibial nerve		
DML, ms	12.7	13.7
CV, m/s	14.7	14.6
CMAP amplitude	0.40	0.28
TLI	0.536	0.500
Peroneal nerve		
DML, ms	17.5	16.8
CV, m/s	NR	9.8
CMAP amplitude	0.13	0.11
TLI	NR	0.486
SENSORY		
Ulnar nerve		
SNAP	NR	NR
CV	NR	NR
Median nerve		
SNAP	NR	NR
CV, m/s	NR	NR
Superficial peroneal nerve		
SNAP	NR	NR
CV, m/s	NR	NR

ARAs, anti-rituximab antibodies; DML, distal motor latency; CV, conduction velocity; CMAP, compound muscle action potential; TLI, terminal latency index; SNAP, sensory nerves action potentials; ND, not done; NR, No response.

### 3.4 Treatment therapy and ARAs detection

**Figure 3:**
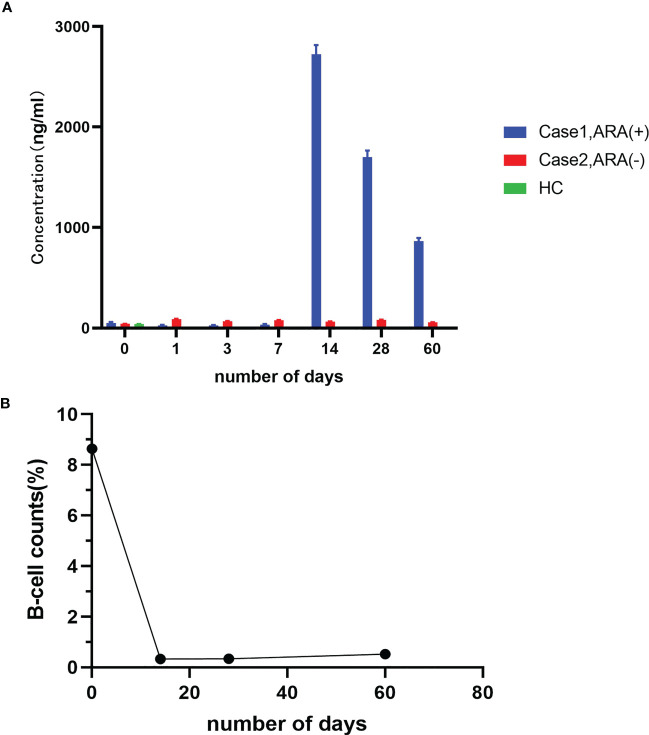
Detection of ARAs and peripheral CD19^+^ B-cell counts. **(A)** ARAs in case 1 and case 2 at admission and on days 1, 3, 7, 14, 28, and 60 after the final round of rituximab infusion; **(B)** Peripheral CD19^+^ B-cell counts in case 1 at admission and on day 1, 3, 7, 14, 28, and 60 after the final round of rituximab infusion.

After the final round of rituximab treatment (500 mg, IV) was prescribed, the CD19^+^ B cell counts were 8.64%, 0.33%, 0.34%, and 0.52% before treatment and on days 14, 28, and 60, respectively. However, there was no significant improvement in clinical features, and △Hughes scored 0. No adverse events associated with rituximab administration were observed. No elevated ARAs titer was detected prior to the final rituximab treatment or on days 1, 3, and 7 after rituximab administration. However, high ARAs titer was detected on day 14, gradually decreasing on days 28 and 60 but remaining higher than normal. In contrast, the other anti-NF155 nodopathy patient had a favorable response to rituximab and did not have a detectable ARAs titer at various time points during the 2 months after rituximab treatment (**Figure 3**).

## 4 Discussion

Our study is the first to report the occurrence of ARAs in patients with anti-NF155 antibodies. We found that ARAs can occur in patients with anti-NF155 nodopathy after rituximab treatment. The poor clinical response to rituximab treatment observed may be associated with ARAs without rapid peripheral CD19^+^ B cell augmentation in anti-NF155 nodopathies, thus ARAs testing early during intervention are encouraged, especially for those showing a poor response to rituximab. Rituximab is a chimeric monoclonal antibody containing human IgG1 constant regions and a murine anti-human CD20 variable region that can lyse CD20^+^ cells (17). It has demonstrated sufficient efficacy and good tolerability in many autoimmune diseases. Rituximab also induced clinical remission in approximately 80% of patients with anti-NF155 nodopathy both at common doses (375 mg/m^2^ weekly for 4 weeks followed by additional doses) and low doses (0.1 g and 0.5 g, IV) (4, 6, 7). However, rituximab can elicit anti-drug antibodies due to the high immunogenicity resulting from its structure. In fact, antibodies targeting cell membrane-linked molecules may have a higher risk of immunogenicity compared with antibodies targeting soluble molecules (18, 19). Since CD20, the target antigen of rituximab, is present on the B cells membrane, rituximab could bind to the target antigen and quickly be internalized along with it (20). The occurrence of ARAs following rituximab treatment has been described in malignancies such as CD20^+^ B-cell non-Hodgkin’s lymphoma and chronic lymphocytic leukemia, and many autoimmune diseases such as RA, SLE, CIDP, multiple sclerosis (MS), membranous nephropathy (MN), pemphigus, and steroid-dependent nephrotic syndrome (SDNS) with highly variable frequencies, ranging from 2.7%–38.46% (11, 12, 14, 21–27). Many factors, such as the number and frequency of infusions, age and gender, product type, and B cell number, might influence ARAs production. These factors may vary in different diseases (27). For example, the number and frequency of rituximab injections are likely to affect the formation of ARAs in MS but not in lymphoma, leukemia, or Crohn’s disease (23, 28, 29). Moreover, ARAs development was associated with reduced B-cell depletion in rituximab-treated patients in MS, but this association is controversial in SLE (23, 30, 31). Therefore, the factors inducing ARAs formation in nodopathy should be deeply explored.

ARAs can somewhat impair therapeutic efficacy by neutralizing rituximab activity, as the ARA-positive group had lower rituximab levels after rituximab infusion compared to the ARA-negative group in autoimmune diseases such as RA and SDNS (13, 21, 25). Therefore, we assumed that the poor outcome of anti-NF155-positive patients who did not respond well to rituximab might be attributed to the occurrence of ARAs. Besides, ARAs in patients with SLE can neutralize drug level and counteract the cytotoxicity of rituximab *in vitro* (31). In nodopathy, the functional assessment of ARAs and drug level are worthy of deep exploration and research.

In our anti-NF155-positive patient, the △Hughes score and all clinical symptoms, including muscle weakness, numbness, and tremor, did not improve after ARAs formation. This indicates that ARAs may also have an impact on the efficacy of rituximab in patients with autoimmune nodopathy. In fact, previous studies have revealed that ARAs can attenuate the efficacy of rituximab (23). However, the effect of ARAs varied among diseases. Specifically, some reported that ARAs correlate with impaired normalization of double-stranded DNA titers in SLE and the requirement for increased frequency of rituximab reinfusion to maintain clinical response in NMOSD (8, 9). Other studies have demonstrated that ARAs affect the relapse rate in MN and SDNS (14, 22). Therefore, further research is needed to explore the association between ARAs and treatment outcomes in anti-NF155 nodopathy. Moreover, the patient achieved favorable outcomes after the first round of rituximab, indicating that rituximab has a response to anti-NF155 nodopathy. ARAs were identified when the patient did not have a satisfied efficacy after the final round of rituximab treatment further confirmed the function. Anti-NF155 IgG4 antibodies are pathogenic as they prevent paranodal complex formation *in vivo* (32). Since rituximab is regarded as a monoclonal antibody against CD20, we suppose anti-NF155 IgG4 antibodies are produced by CD20^+^ antibody-secreting cells (ASCs). Indeed, these CD20^+^ ASCs mainly consist of short-lived plasma cells instead of long-lived plasma cells that most immunoglobulins are produced by (33–35).

However, the association between the presence of ARAs and incomplete B-cell depletion is controversial. Specifically, there was a significant association between ARAs’ presence and titers and incomplete B-cell depletion in MS, and the ARA-positive group had a faster B-cell reconstitution in SDNS and MN (14, 22). The count of CD19^+^ B cells after rituximab infusion in the ARAs group in SLE varied as Chris Wincup showed that no difference was found in CD19^+^ lymphocyte counts at the early and six-month time points between ARAs persistently positive and negative patients and Francesca Faustini reported that a higher proportion of CD19 ^+^ lymphocytes was seen in ARA-positive patients compared to ARA-negative patients (31, 36). Moreover, most patients with MOGAD had a B-cell counts <1% at the time of disease activity (37) and B-cell counts have been found to be unrelated to clinical relapse in NMOSD (38). Therefore, whether the low count of CD19^+^ B cells within 2 months after our patient’s last round of rituximab treatment is due to the short duration between rituximab treatments and the point of ARAs detection or due to the nature of anti-NF155 nodopathy needs more cases and follow-up time to explore. And potential mechanisms of non-autoantibody mediated damage in autoimmune nodopathy should also be explored deeply in further studies. At least, our case may indicate that CD19^+^ B cell count is not associated with clinical outcome or ARAs formation within 2 months.

The side effects of rituximab infusion mainly included serum sickness, skin rash, and other infusion-related reactions (8, 22). In our study, the anti-NF155-positive patient did not show any side effects of rituximab. Whether the frequency of the adverse events was associated with ARAs titers or not is controversial among previous studies (8, 13, 22, 23). Further research, with larger sample sizes, is needed to indicate the side effects of rituximab in ARA-positive autoimmune nodopathy.

Compared to half-human half-murine rituximab, the completely humanized anti-CD20 monoclonal antibodies such as ocrelizumab (OCR) and ofatumumab have lower immunogenicity and less frequency of anti-drug antibody occurrence. These completely humanized anti-CD20 monoclonal antibodies are well-tolerated and had favorable outcomes in many patients with autoimmune diseases such as CIDP, MN, SLE, and MS (8, 10, 12, 14, 22, 23, 39). However, in these cases, OCR or ofatumumab treatment was preceded by rituximab treatment and prescribed after the development of ARAs. As ARAs cannot inhibit the cytotoxicity of humanized anti-CD20 monoclonal antibodies, we suggest that new humanized anti-CD20 antibodies might be considered a potential therapeutic alternative in anti-NF155 nodopathy (14). Immunosuppressants could also be tried in ARA-positive anti-NF155 nodopathy as they affected part of patients with anti-NF155 nodopathy (40).

There are several limitations to this study. First, the level of serum rituximab was not detectable in our case; therefore, the association between the pharmacokinetics of rituximab and ARAs cannot be analyzed. Second, ARAs were not tested after the 1st-4th rituximab infusion as the patient has not been treated in our institution before the last infusion, and no blood samples were available during that time. Since anti-NF155 nodopathy is a rare disease and incidence of these patients with poor rituximab response is lower (7), the size of our sample of anti-NF155-positive patients with ARAs was limited. Thus, more studies are needed to explore the association between ARAs and B cell counts, clinical efficacy, and allergic reactions in patients with anti-NF155 nodopathy.

In conclusion, this is the first case to report the occurrence of ARAs in patients with anti-NF155 antibodies. We found that ARAs may be produced in patients with anti-NF155 nodopathy after rituximab treatment and attenuate rituximab efficacy without adverse effects. It is important to raise our awareness of ARAs to adjust the treatment strategy for autoimmune nodopathy patients who received rituximab but had poor outcomes. We suggest that an early testing of ARAs during rituximab intervention will be beneficial for a more individualized approach to patient. And we also believe that the new humanized anti-CD20 monoclonal antibody might serve as a promising therapeutic alternative in anti-NF155 nodopathy, but further studies are needed to confirm this in the future.

## Data availability statement

The raw data supporting the conclusions of this article will be made available by the authors, without undue reservation.

## Ethics statement

The studies involving human participants were reviewed and approved by the Ethics Committee of Qilu Hospital. The patients/participants provided their written informed consent to participate in this study. Written informed consent was obtained from the individual(s) for the publication of any potentially identifiable images or data included in this article.

## Author contributions

Study concepts, patient evaluation, data acquisition, interpretation and analysis, and manuscript drafting: YB. Study design and data acquisition: WL and CY. Anti-NF155 testing and manuscript revision: YH and WL. Study design, interpretation of results, and manuscript revision: QW. All authors have contributed to the manuscript and approved the submitted version.
